# Correlating NAD(P)H lifetime shifts to tamoxifen resistance in breast cancer cells: A metabolic screening study with time-resolved flow cytometry

**DOI:** 10.1142/s1793545824500202

**Published:** 2025-01

**Authors:** Samantha Valentino, Karla Ortega-Sandoval, Kevin D. Houston, Jessica P. Houston

**Affiliations:** * Chemical and Materials Engineering, New Mexico State University 1040 S Horseshoe Dr., Las Cruces, NM 88003, USA; † Chemistry and Biochemistry, New Mexico State University 1175 N Horseshoe Dr., Las Cruces, NM 88003, USA

**Keywords:** Time-resolved, flow cytometry, autofluorescence, fluorescence lifetime, breast cancer, metabolism

## Abstract

Time-resolved flow cytometry (TRFC) was used to measure metabolic differences in estrogen receptor-positive breast cancer cells. This specialty cytometry technique measures fluorescence lifetimes as a single-cell parameter thereby providing a unique approach for high-throughput cell counting and screening. Differences in fluorescence lifetime were detected and this was associated with sensitivity to the commonly prescribed therapeutic tamoxifen. Differences in fluorescence lifetime are attributed to the binding states of the autofluorescent metabolite NAD(P)H. The function of NAD(P)H is well described and in general involves cycling from a reduced to oxidized state to facilitate electron transport for the conversion of pyruvate to lactate. NAD(P)H fluorescence lifetimes depend on the bound or unbound state of the metabolite, which also relates to metabolic transitions between oxidative phosphorylation and glycolysis. To determine if fundamental metabolic profiles differ for cells that are sensitive to tamoxifen compared to those that are resistant, large populations of MCF-7 breast cancer cells were screened and fluorescence lifetimes were quantified. Additionally, metabolic differences associated with tamoxifen sensitivity were measured with a Seahorse HS mini metabolic analyzer (Agilent Technologies Inc. Santa Clara, CA) and confocal imaging. Results show that tamoxifen-resistant breast cancer cells have increased utilization of glycolysis for energy production compared to tamoxifen-sensitive breast cancer cells. This work is impacting because it establishes an early step toward developing a reliable screening technology in which large cell censuses can be differentiated for drug sensitivity in a label-free fashion.

## 1. Introduction

Breast cancer is one of the most common cancers diagnosed worldwide.^1^ In 2023, it is projected that approximately 297,790 cases of invasive breast cancer will be diagnosed in women in the United States, a sustained 0.5% increase in incident diagnoses per year.^2^ In the United States the average risk of women developing breast cancer is 13%, or one in eight, and in 2023 it was estimated that approximately 43,700 women will die of breast cancer.^2^ Moreover, there are numerous complications associated with breast cancer that inhibit prognosis and recovery, such as the development of treatment resistance. Resistance to therapy can occur with different drugs and combinations thereof. One in particular is the chemotherapeutic tamoxifen.

Tamoxifen is a hormone therapy used for all stages of estrogen receptor (ER)-positive breast cancer in both men and women. Originally developed in the 1960 s as a contraceptive, tamoxifen eventually became a leading treatment for ER-positive breast cancer and the first available preventative care drug.^3,4^ Despite the undeniable benefits of tamoxifen and its wide treatment potential, it is limited by susceptibility to resistance, typically acquired within 2 to 5 years of treatment.^5^ It is estimated that one-third of women treated with tamoxifen for 5 years will be diagnosed with recurrent disease within 15 years.^6^ Owing to the prevalence of tamoxifen resistance, we and others have focused on understanding the mechanisms of tamoxifen resistance, specifically how antiestrogenic tamoxifen action is altered within estrogen-dependent signal transduction pathways.^6–11^

In this contribution, we present new results that add to a holistic picture of the etiology of tamoxifen resistance. We have specifically focused on cellular respiration because of the varied evidence showing how cellular metabolism changes in malignant cancers. Many cancers maintain high rates of glycolysis in the presence of oxygen,^5,12^ and some cells exhibit distinct metabolic phenotypes that occur in response to oncogene activation.^5^ In breast cancer, altered metabolism has been observed *in vitro*, and is varied depending on if the cell type is normal, cancerous, or exposed to therapeutics.^13,14^ Additionally, tamoxifen treatments given to tamoxifen resistant MCF-7 cells (i.e., estrogen receptor positive breast cancer cells) increase lactate production, which is associated with glycolysis.^15,16^ Tamoxifen resistance has also been correlated to mitochondrial complex I inhibition, decreased oxygen consumption, and decreased ATP production, which directly points to a preference for glycolysis.^17–19^ Increased AMPK and AMPK phosphorylation have also been observed and associated with an increased ratio of AMP/ATP.^17^ While these findings have been associated with promoting glycolysis and altered fatty acid metabolism, which can be used to replenish ATP levels during metabolic stress within resistant populations, there are conflicting ideas on the proposed methodology of oxidative phosphorylation (OXPHOS) inhibition in the literature.^17,20^ Metabolomic and proteomic analyses also reveal a different profile of metabolites in MCF-7 cells depending on whether they are tamoxifen-treated or not, and with resistance.^14,21^ The fact that metabolism is clearly altered upon treatment with tamoxifen by an undefined mechanism indicates there is a clear opportunity to explore if metabolism is associated with resistance development.

### 1.1. Background

We focus herein on a quantitative approach for observing metabolic pathway dynamics (transition between oxidative phosphorylation and glycolysis) to classify cultured breast cancer cells based on metabolic phenotype. The metabolic phenotype is quantified using autofluorescence as a unique biomarker. Reduced nicotinamide adenine dinucleotide (phosphate) (NAD(P)H) is autofluorescent and is a metabolic cofactor that can be used for quantitative metabolic profiling.^22^ It consists of the combined fluorescence of NADH and NADPH which have identical absorption and emission and are therefore collectively measured as NAD(P)H.^23^ NAD(P)H is an electron/proton carrier involved in glycolysis followed by cytosolic lactic acid fermentation and OXPHOS in the mitochondria.^5,22^ Redox reactions with NAD(P)H result in the conversion of ADP to ATP, and the reactions depend on the action of NAD(P)H during glycolysis or OXPHOS.^12,22,24–26^

Owing to its role in metabolism, NAD(P)H has been optically characterized for decades.^27^ When excited at its optimal excitation wavelength, the emission intensity of NAD(P)H varies depending on its reduced or oxidized form, location inside of cells (e.g., mitochondria, cytosol), and the local intracellular pH.^28^ Thus, endogenous fluorescence of NAD-(P)H is a favorable biomarker of metabolism.^28–30^ However, evaluating differences between cells based on autofluorescence brightness is challenging since autofluorescence signals are proportional to concentration and affected by instrumentation artifacts (i.e., signal-to-noise). The fluorescence lifetime is a photophysical trait independent of autofluorescence brightness, and conveniently, NAD(P)H when excited, exhibits a two-component fluorescence lifetime.^24^ Therefore, the bi-exponential fluorescence decay of NAD(P)H can be used to study cellular energetics by reflecting the state of the cofactor. NAD(P)H binds to catalytic substrates (e.g., dehydrogenases like malate, lactate, isocitrate, succinate) during redox reactions, and when “free” exhibits a shorter lifetime component (0.3–0.8 ns) compared to a longer decay (1–6.5 ns) when bound.^31^ This binding has been associated with several enzymes that influence this broad range of values for the fluorescence lifetime.^32^ The fluorescence lifetime of NAD(P)H has also been associated with the enzymatic conformational changes that occur with binding to the NADH or NADPH cofactor and the resulting fluorescence lifetimes.^23^ In general, an increase in the long-lifetime component of NAD(P)H is an indicator of the shift of the cellular metabolism from glycolysis to OXPHOS.^22,24,25^

Our work presents a unique cytometry approach that screens for shifts in the fluorescence lifetime of NAD(P)H thereby providing a way to characterize the bioenergetics of large populations of breast cancer cells. We have developed time-resolved flow cytometry (TRFC) systems that measure fluorescence lifetimes and generate parameters based on the fluorescence dynamics of fluorophore-tagged cells at rates of ~1000 s cells/s.^33–36^ The throughput of TRFC is advantageous over fluorescence lifetime imaging microscopy (FLIM), when it is anticipated that the large cell population is heterogeneous or if rare cell phenotypes are present. Prior examples of TRFC include cell sorting based on the fluorescence lifetime,^29,37^ development of phasor data,^38^ microfluidic platforms,^39–41^ time-domain platforms,^35,42,43^ alternative focusing methodologies with acoustics,^44^ and many applications thereof which have been well summarized in a review from Houston *et al.*^45^ As mentioned previously, metabolic changes in chemotherapeutically treated and resistant breast cancer cells are complex. Thus, our study seeks to determine if TRFC reveals differences in the fluorescence lifetime of NAD(P)H between tamoxifen resistant and sensitive cells.

It is important to note that recent advances in high-throughput FLIM are comparative to TRFC as a cell analysis technology, yet provide different advantages — primarily that being imaging. For example, single photon avalanche diode (SPAD) detectors improve the spatial and/or temporal resolution and FLIM speed.^46–49^ This has been demonstrated as well with spectro-temporal laser imaging by diffracted excitation (SLIDE), which can acquire up to 2000 frames per second.^45,50^ Additionally, two- and multi-photon FLIM studies of T lymphocyte metabolism^51^ and optical redox ratio measurements of B cells,^52^ respectively, have shown promise for higher throughput imaging. Others have been outlined in a more comprehensive review by Datta *et al.*^53^

In this work, we focus on the ability to measure shifts in the fluorescence lifetime during chemotherapeutic resistance and thereby evaluate the limits and confines of TRFC performance. We use a subset of data detectable at a throughput of 100s of cells/s due to dim autofluorescence limitations to calculate the fluorescence lifetime and compare the subtle differences between sensitive and resistant cancer cells. This work sets the stage for real-time autofluorescence lifetime cytometry sorting and screening for molecular signatures of drug resistance.

## 2. Materials and Methods

Our methods included a comprehensive characterization of the metabolic profile of breast cancer cells resistant to and sensitive to the therapeutic tamoxifen. We evaluated cells with TRFC, Seahorse analyses, and fluorescence microscopy for cell-to-cell autofluorescence. The details of these methods are provided below.

### 2.1. Time resolved flow cytometry and fluorescence lifetime analysis

**Fig. 1.**
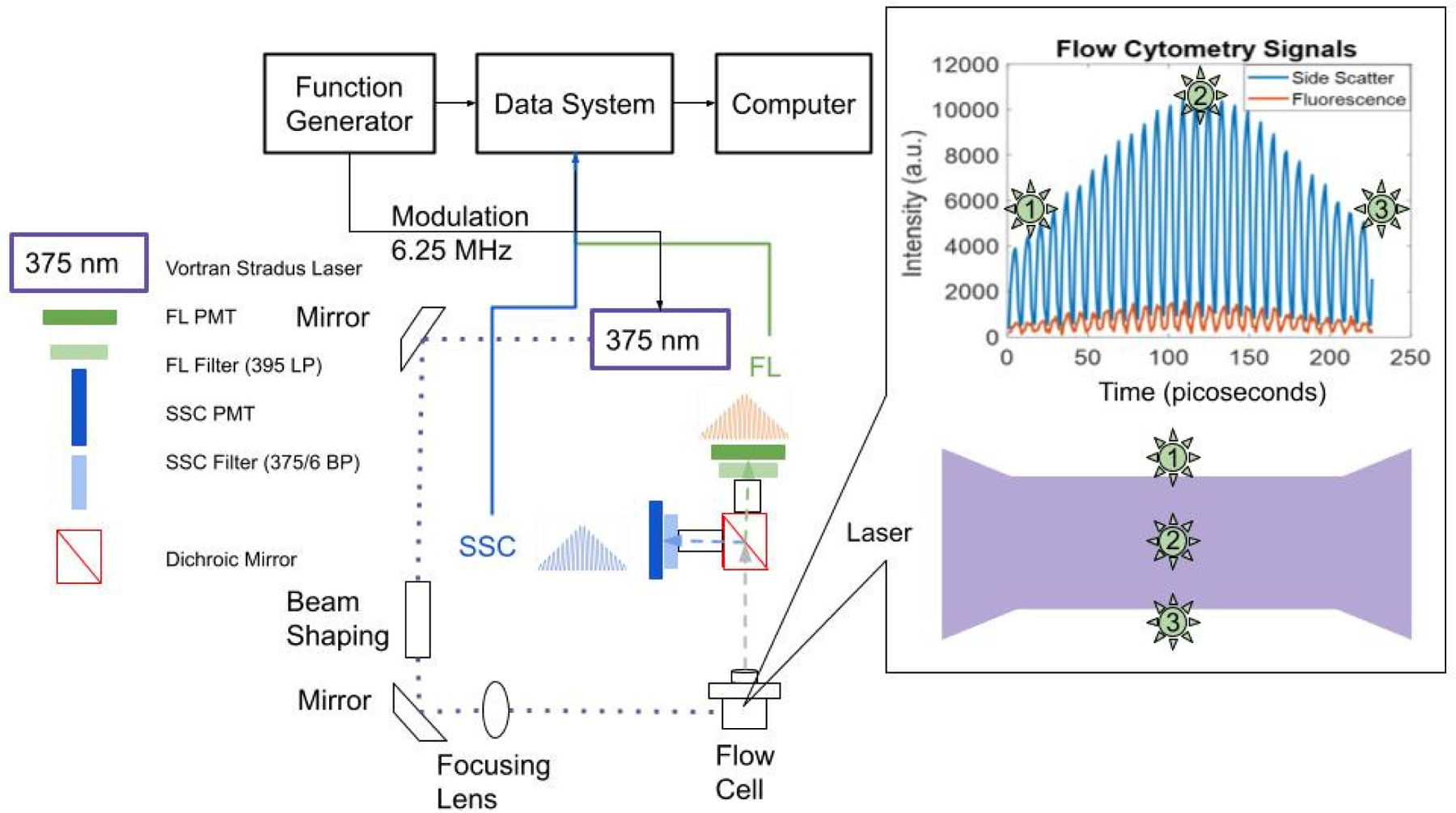
Schematic of the time-resolved flow cytometer. Cells are hydrodynamically focused and passed through a 375 nm laser. The movement of the cells is illustrated: point 1 is the position of the cell as it enters the laser, point 2 is the time when it is located in the center of the laser, and point 3 is where the cell leaves the laser region. The cell transit times are ~10 *μ*s. The resulting light scatter and emission are illustrated. Light is filtered using a 375/6 BP filter for side scatter detection (depicted in blue). The fluorescence detector is depicted in green, and collects emission light through a 395 LP filter.

The fluorescence lifetime is defined as the average time a fluorophore spends in the excited state before returning to the ground state.^29^ The fluorescence lifetime when calculated using a frequency-domain approach is mathematically defined as follows:

$$\tau =\frac{\tan(\text{Δ}\varphi )}{2*\pi *F},$$

where τ is the fluorescence lifetime, F is the modulation frequency, and Δφ represents the phase shift between the frequency-modulated excitation and emission signals. With time-resolved flow cytometry (TRFC) the phase shift is calculated as the difference between the side scattered light signal phase (SSC) and fluorescence (FL) phase. TRFC is schematically presented in Fig. 1 and has been described thoroughly in other contributions.^29,30,33,54–56^ Cells are measured individually as they rapidly pass through a laser excitation source. Cell alignment is achieved by hydrodynamic focusing with DI water as the sheath fluid. A 375 nm 60 mW (Vortran Stradus^®^) laser is used to excite internal NAD(P)H within the cultured and treated cells (see methods below). The fluorescence and scattered light from each cell are detected with photomultiplier tubes (PMT) (Hamamatsu, H10720-20 (side scatter) and H10720-210 (fluorescence)), with a peak sensitivity of 630 nm and 400 nm, respectively. A 375/6-nm band pass optical filter was selected for side scatter detection and a 395-nm long pass for fluorescence emission of NAD(P)H. For frequency domain data collection, a function generator (MSO5000, Rigol) was used for laser modulation at 6.25 MHz. A custom data acquisition system with a sampling rate of 50-MSPS was used for waveform readout. Thus, our modulation frequency was chosen such that the digitization rate is greater than twice the frequency to satisfy the Nyquist sampling theorem. The resulting “cytometric waveforms” were saved and used for processing (see Fig. 1).

### 2.2. Time-resolved flow cytometry calibration and SNR

**Fig. 2.**
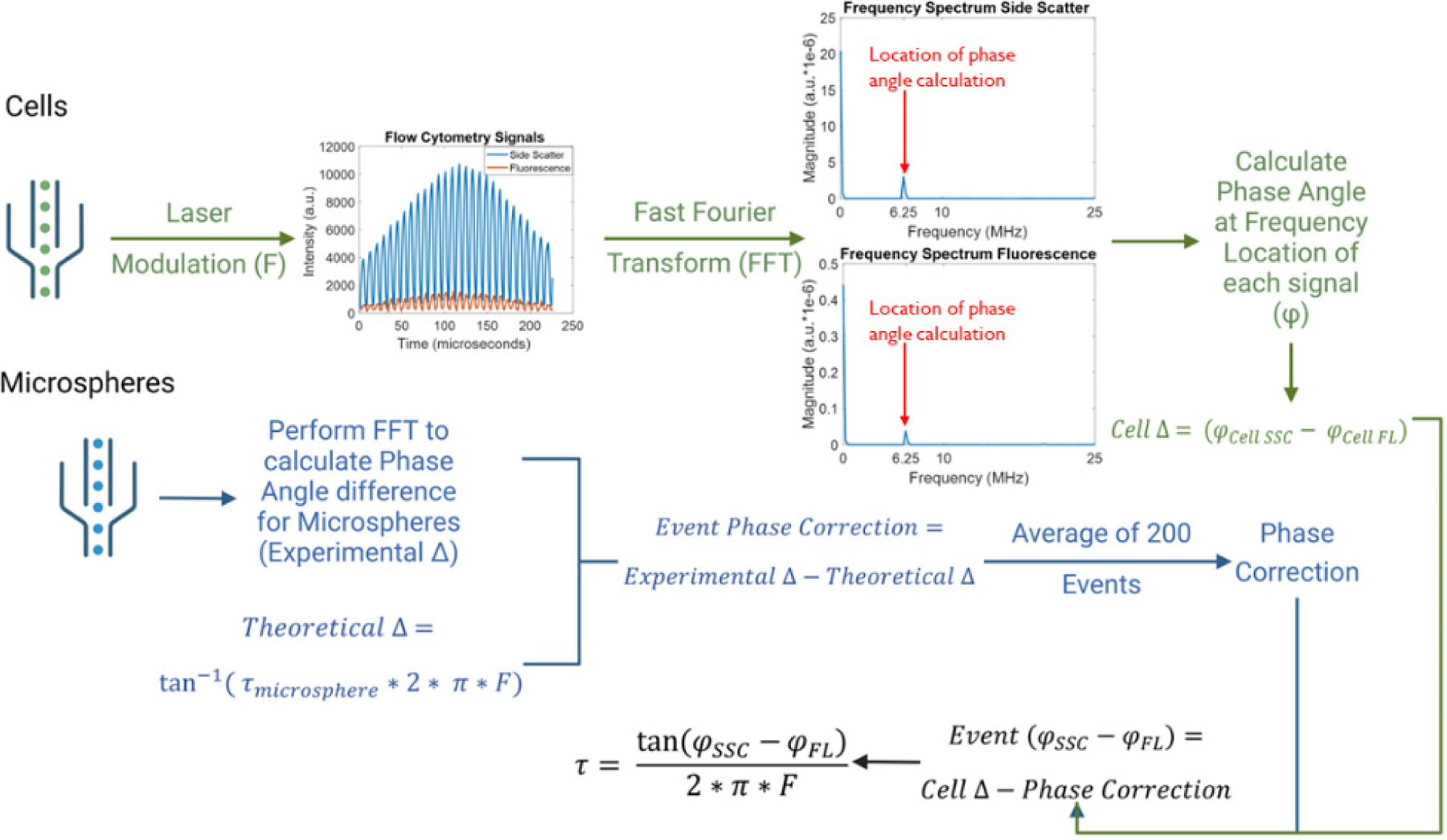
Workflow for the calculation of average fluorescence lifetimes per cell/microsphere. System settings were established to first optimize the signal-to-noise. Microspheres were evaluated to find a phase difference based on 200 events. Then, a theoretical phase difference was calculated by correcting the measured phase based on a known fluorescence lifetime (7-ns). That is, we found the difference between the measured phase shift and the phase shift that renders a 7-ns lifetime. After this, cells with unknown fluorescence lifetimes were measured based on this correction. Fast Fourier Transform (FFT) analyses were performed to determine phase values. Image created with BioRender.com.

**Fig. A.1.**
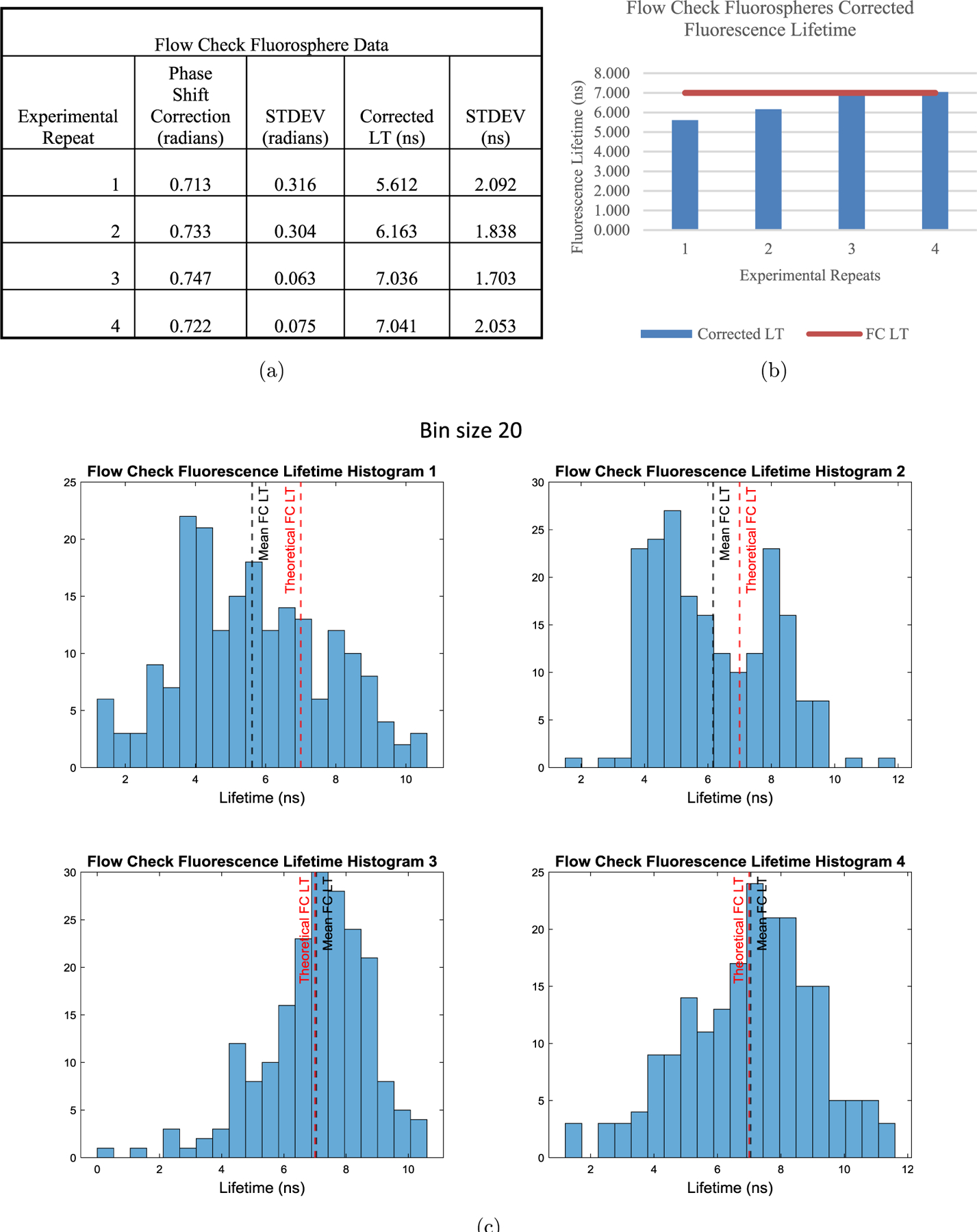
The Flow-CheckTM fluorosphere data collected from each of the four experimental repeats is presented in the above figures. (a) This table presents the phase shift correction calculated for each experimental repeat, as well as the standard deviation of the phase shift for the 200 fluorescent microspheres used for the calculation of the phase shift. This table also shows the fluorescence lifetime calculated for the microspheres following calibration as well as their standard deviation. Successful calibration is ranked based on closeness to the experimental fluorescence lifetime of Flow-CheckTM Pro Fluorospheres to 7 ns. (b) The corrected fluorescence lifetimes of the four experimental repeats of the 200 fluorescent microspheres is presented. The theoretical fluorescence lifetime of Flow-CheckTM Pro Fluorospheres is presented for reference within the figure. (c) The 200 fluorescent microsphere corrected fluorescence lifetime histograms from the four experimental repeats is presented and the theoretical fluorescence lifetime is marked at 7 ns.

Calibration of our time-resolved measurements was performed using fluorescence microspheres (Flow-Check^™^ Pro Fluorospheres, Beckman Coulter Life Sciences, #A63493). These microspheres are imbibed with different fluorophores for broad excitation and emission. Flow-Check^™^ Pro Fluorospheres also have a unique and stable fluorescence lifetime, reported in previous literature to be 7 ns.^33,56^ Calibration is shown schematically in Fig. 2. First, cells were measured to establish baseline instrumentation settings (gains, flow rates, and data acquisition parameters). Subsequently, fluorescent microspheres were suspended at a concentration of 1× 10^6^ microspheres/mL DI water and measured with the TRFC system. Microsphere waveforms were collected holding all TRFC settings the same. We next used the microsphere waveforms to determine a reference phase shift based on the fluorescence lifetime value of 7 ns by back-calculation of Eq. (1). That is, un-corrected phase shifts $\left(\varphi_{SSC}-\varphi_{FL}\right)$ using microsphere waveform data were obtained, then averaged. The average was corrected for a phase shift rendering a 7-ns lifetime. Following this process, cells were analyzed, and cytometric waveforms were collected. The average fluorescence lifetime was then calculated relative to the phase correction using 200 events (i.e., cells) that met the three criteria described below. TRFC can read out the fluorescence lifetimes as standard cytometry parameters and standard event rates. Yet we chose to evaluate a small number of waveforms offline (200). This is because of the inherent noise present with data sets from dim signals and because of data acquisition restrictions (i.e., Nyquist modulation frequency = 25 MHz). Thus, we selected the subset of waveform data (i.e., 200 of > 1000 events) that met these criteria: (1) waveform had adequate signal-to-noise ratios; (2) waveform was not truncated (had adequate modulation cycles for FFT analysis); and (3) data sets could be batch-processed in near-real time. Figure A.1 includes data to support this methodology and principles of this correction are presented in our prior published work.^56^

We evaluated signal levels of the TRFC to understand the instrumentation limitations when attempting to measure NAD(P)H autofluorescence at standard throughputs. Our approach included signal-to-noise ratio (SNR) calculations where we first evaluated the noise floor. We held all system component settings the same as when cells were being counted. These include the laser power, PMT voltages, modulation frequency, and data acquisition software settings. The inherent noise of the TRFC was then assessed by using a software trigger on the noise threshold. These values were digitized as ‘pseudo-waveforms’ and represented baseline signals no cells are actively being measured. 200 pseudo-waveforms were evaluated for maximum intensity and the average maximum intensity of the noise. Subsequently, cells were analyzed. The average maximum intensity from cells was taken as the signal. This was done for four independent experiments. This average intensity was compared to the noise as follows:

$$\text{SNR}=\frac{\text{Average maximum intensity of MCF}-7\text{ or MCF}-7\text{ TamR}}{200\text{ pseudo event average maximum intensity of noise}}.$$

This was completed for both the MCF-7 tamoxifen-sensitive cells $\left(\text{MCF-}7\right)$ and MCF-7 tamoxifen-resistant cells $\left(\text{MCF-}7\text{ TamR}\right)$. The resulting signal-to-noise ratio was 7 for the MCF-7 cells and 9 for the MCF-7 TamR. Additionally, we performed a statistical photon count based upon the work of Giesecke *et al.*^57^ Data are included in Table D.1. The number of photons detectable provides an additional metric for the sensitivity of autofluorescence detection by this TRFC system. This was an approximation because the method is designed for standard cytometry (i.e., non-modulated waveforms). Lastly, we calculated the laser light fluence in order to understand the total power impacting the cell. We estimate that it is 6:04 × 10^−10^ J/cm^2^ as defined in Table E.1.^58^

### 2.3. Cell culture conditions and tamoxifen resistance establishment

MCF-7 cultured cell populations were selected for all metabolic analyses to build upon our prior work characterizing how this cell type becomes tamoxifen resistant.^11^ Standard culture conditions were followed which involved maintaining cells at 37°C, 90% humidity, 5% CO_2_ and with high glucose DMEM (Gibco^™^, Catalog #31053028, 4.5 g/L D-Glucose), 1X Sodium Pyruvate (Gibco^™^, Catalog #11360070, 100 mM 100X), 1X L-Glutamine (Gibco^™^, Catalog #25030081, 200 mM 100X), and 10% Fetal Bovine Serum (Gibco^™^, Catalog #A4766801, Fetal Bovine Serum). Cells were cultured to approximately 90% confluency prior to collection and analysis. We established resistant subpopulations by continually maintaining cells in 1 *μ*M tamoxifen within their growth medium. Tamoxifen was diluted in ethanol to a concentration of 1 mM and this solution was added to cell maintenance media for treatment of 1 *μ*M tamoxifen. Over time the cells maintained and sustained proliferation in the presence of tamoxifen and were established as a resistant subpopulation of MCF-7 cells. Tamoxifen resistant subpopulations are referred to as MCF-7 TamR cells for the remainder of this paper. Cells were prepared for cytometry measurements by detaching from the flask using trypsin-EDTA (Gibco^™^, Catalog #25200056, Trypsin-EDTA (0.25%)), resuspending in culture media, centrifugation and resuspension in 1X PBS (phosphate buffer saline) at a concentration of 1 × 10^6^ cells/mL. Cells were measured on the TRFC within one hour of collection. A Countess^™^ Cell Counter (ThermoFisher Scientific) was used to determine cell count. For TRFC measurement, the cell suspension was run at a velocity of 0.01–0.05 mL/min to allow for an event rate capture of approximately 130–180 events/s. From collected events, 200 were analyzed per cell subtype per experiment for the fluorescence lifetime. A one-way ANOVA was applied to compare the difference between the MCF-7 and TamR fluorescence lifetime across the four independent experiments.

### 2.4. Agilent seahorse HS mini ATP flux rate assay

A real-time ATP rate assay protocol was used for metabolic analysis with the Seahorse XF Real-Time ATP Rate Assay Kit (Agilent Technologies, Inc., Product #103591–100). To conduct these tests 20,000 MCF-7 and MCF-7 TamR cells were plated in separate wells per 80 *μ*L of media in 8-well Seahorse cell culture plates. Cells were plated on the cell plate 48 h before testing to allow for adequate attachment of the cells to the well. Four independent repeats were collected per cell type. A BCA assay was used to normalize protein levels within the wells post-assay.

### 2.5. Autofluorescence microscopy

**Fig. C.1.**
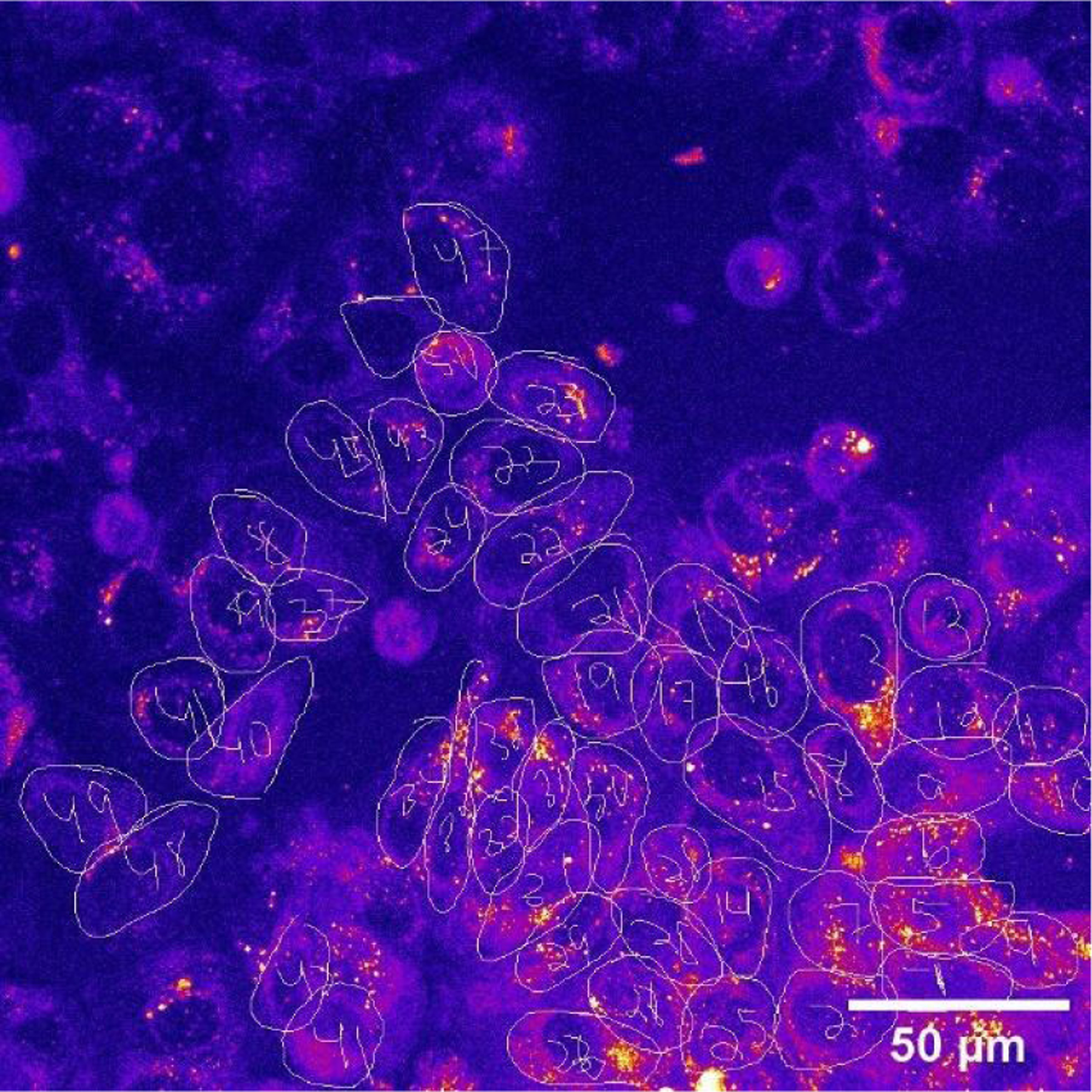
Example of how cells were segregated and evaluated for the raw integrated density using out MCF-7 TamR sample. Images were loaded as hyperstacks into Fiji. A z-stack was then performed on the hyperstacks at maximum intensity to compile the stacks into a single image. Free hand selections were used to segregate out cells and take a measurement of the selected cell. The pencil tool was used to annotate on the image what area had been segregated on the individual cells which is what is presented above, the annotations of the cell areas selected for measurement. Color discrepancies in the pencil tool are due to changes in the look up table that changed the pencil tool color automatically.

Autofluorescence microscopy was conducted to estimate changes in brightness, make observations of morphological differences, and identify any additional features that might indicate cellular heterogeneity. Cells were imaged using a Leica TCS SP5 confocal microscope (Leica Microsystems). To achieve this 5 × 10^5^ cells per dish were plated onto confocal imaging dishes and allowed to culture overnight. For testing a 63X oil-emersion objective was used, and a 405 nm excitation laser source was used to capture the sample images. Emission light was collected from 435 nm to 600 nm at 10% laser power to capture the full autofluorescence spectra upon UV excitation. Results were derived from the average raw integrated density of 50 cells within the field of view. Images were first hyperstacked for visualization in Fiji where a z-stack was then used to combine the z-stacks into a single image. Cells were then individually selected, by eye and the pencil tool within Fiji software (doi:10.1038/nmeth.2019), to calculate the raw integrated density of each cell selected. Various lookup tables were used to enhance the color contrast of the cells for selection. An example of the selection process is in Fig. C.1. The raw integrated density of MCF-7 and MCF-7 TamR cells were then compared. Published images have been subjected to the following enhancements: smoothing, background subtraction with a 50 pixel rolling ball, contrast enhancement to 0.35%, and set contrast min and max to 0 and 28,700, respectively. As previously mentioned, autofluorescence imaging is somewhat arbitrary in that the brightness is a function of many factors. Thus, image processing of intensity averages was performed only to ascertain if a deviation exists between these cell types under the specific aforementioned conditions.

## 3. Results

Metabolic profiling data obtained using TRFC, the Seahorse analyzer, and confocal microscopy, were correlative indicating a distinct metabolic profile for tamoxifen sensitive $\left(\text{MCF-}7\right)$ versus resistant cells $\left(\text{MCF-}7\text{ TamR}\right)$. Cellular respiration in MCF-7 cells was primarily achieved through OXPHOS and glycolysis was utilized to a greater degree within MCF-7 TamR cells.

**Fig. 3.**
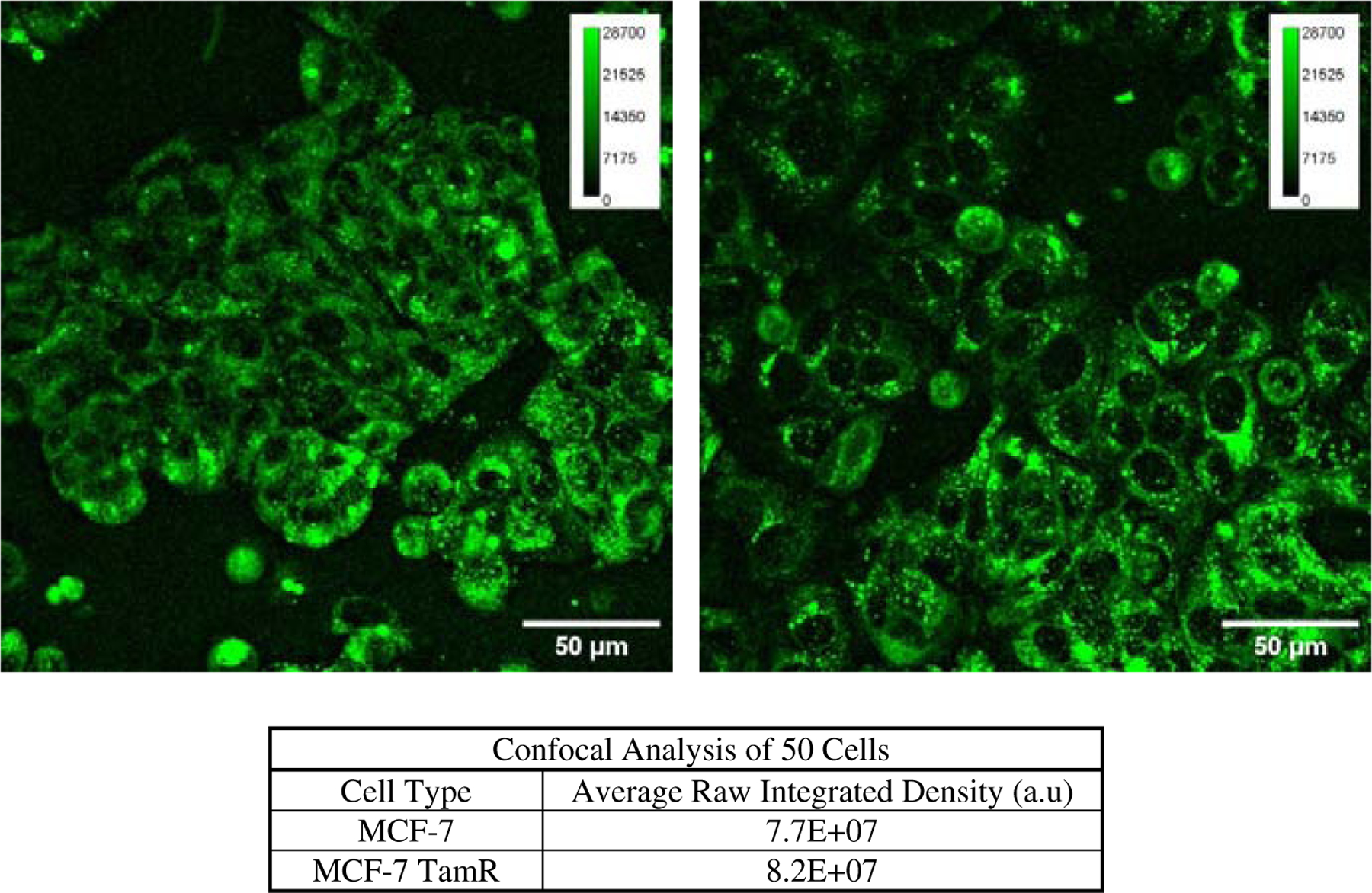
Left: Confocal image of MCF-7 cells. Right: Confocal image of MCF-7 TamR cells. A visual comparison of these images shows there is a brighter NAD(P)H intensity in tamoxifen resistant cells. An analysis of 50 cells from each cell type indicates the NAD(P)H raw integrated density of MCF-7 TamR cells is higher than Parental MCF-7 cells.

Results from confocal NAD(P)H autofluorescence imaging are shown in Fig. 3. The raw integrated density (the sum of the pixel intensities within selected regions of interest), is higher within tamoxifen resistant cells. The autofluorescence microscopy imaging resulted in a higher intensity for the MCF-7 TamR cells, which is similar to other reports of overall brightness. Although nonquantitative, these results do align with Seahorse and TRFC data.

**Fig. 4.**
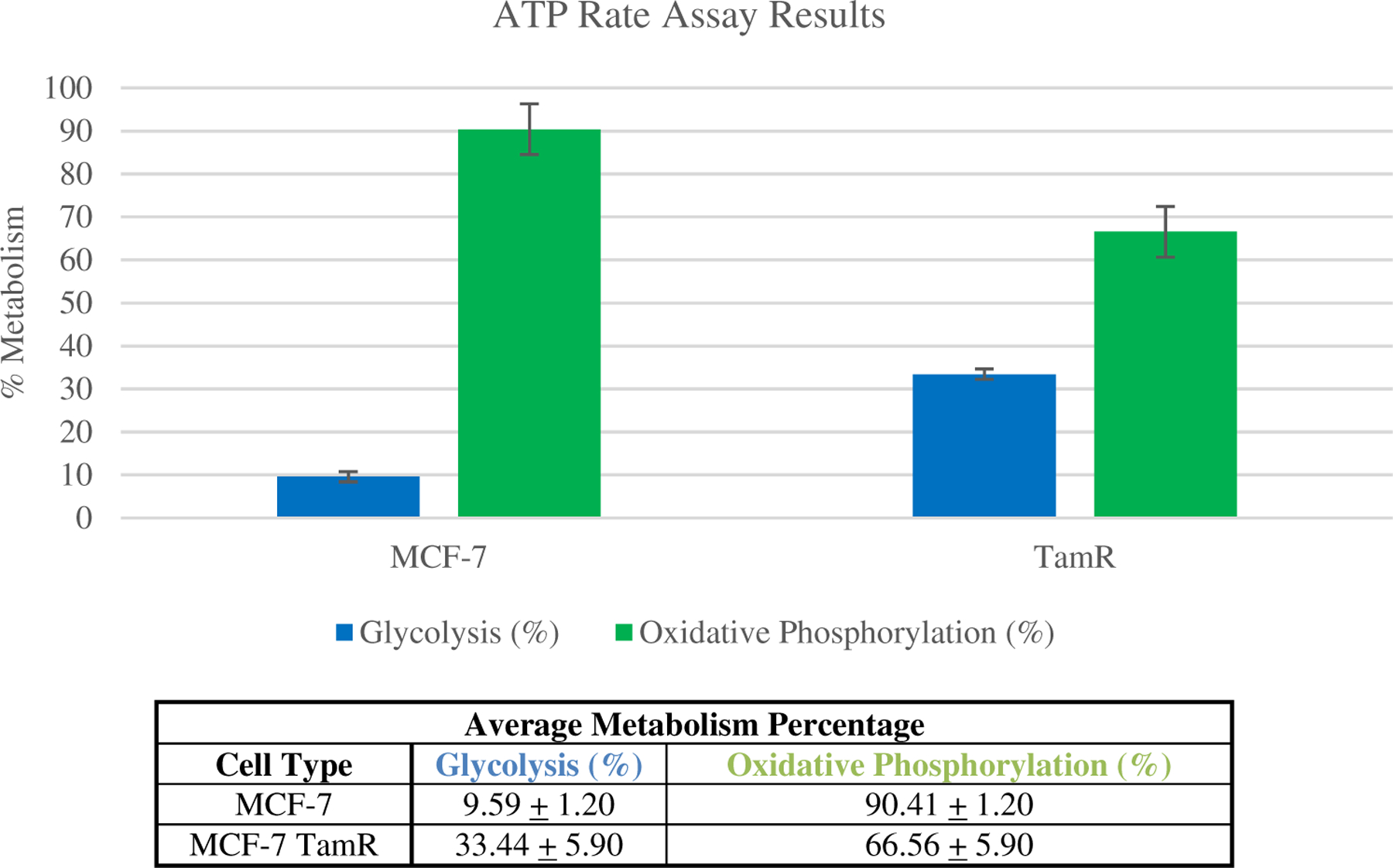
Metabolic cycle influence percentage results for the average Seahorse ATP rate assays for comparing glycolysis (blue) to OXPHOS (green) in tamoxifen sensitive and resistant populations. Table of ATP Flux rate average percent metabolism results of the MCF-7 and MCF-7 TamR results from each of the four experimental repeats.

To establish the differences in the % glycolysis or % OXPHOS measured, the average of four independent cell cultures measured with the Agilent Seahorse HS Mini ATP rate assay is presented in Fig. 4. For MCF-7 cells, there was a 9.59% average influence of glycolysis and 90.41% influence of OXPHOS (standard dev. = 1.20% for both metabolic cycles). The MCF-7 TamR cell measurements resulted in an average 33.44% glycolytic influence and 66.56% OXPHOS (standard dev. = 5.90% for both metabolic cycles). These data indicate a 3.5-fold increase in the reliance of MCF-7 TamR on glycolysis.

**Fig. 5.**
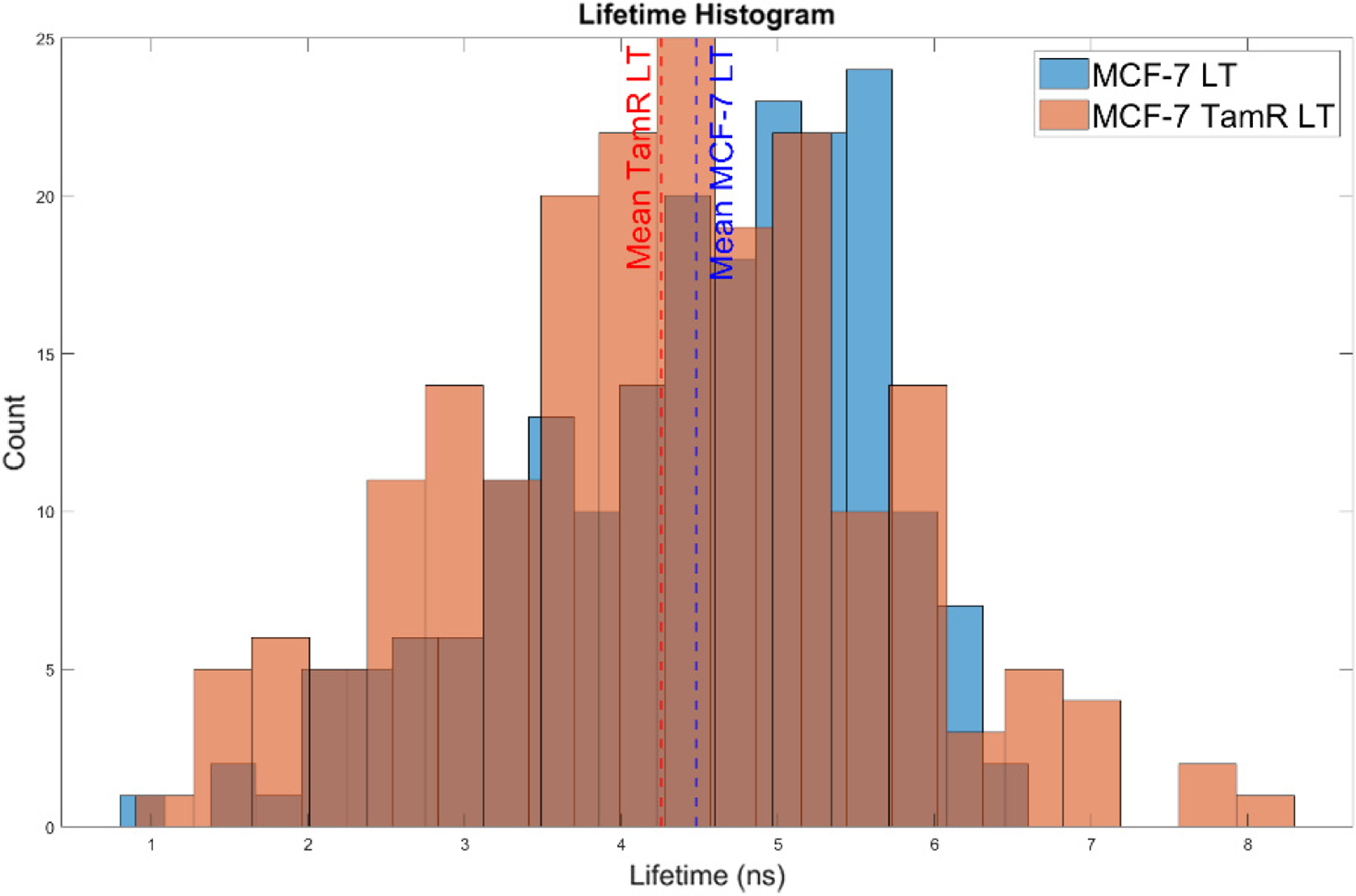
An example histogram of 200 fluorescence lifetimes from MCF-7 (blue) and MCF-7 TamR (orange) cells from a single experiment is presented in the figure. The mean is presented for each of the two cell treatments, the MCF-7 TamR mean in red and the MCF-7 in blue, to show the difference in lifetime found for the two cell treatment subtypes.

**Fig. B.1.**
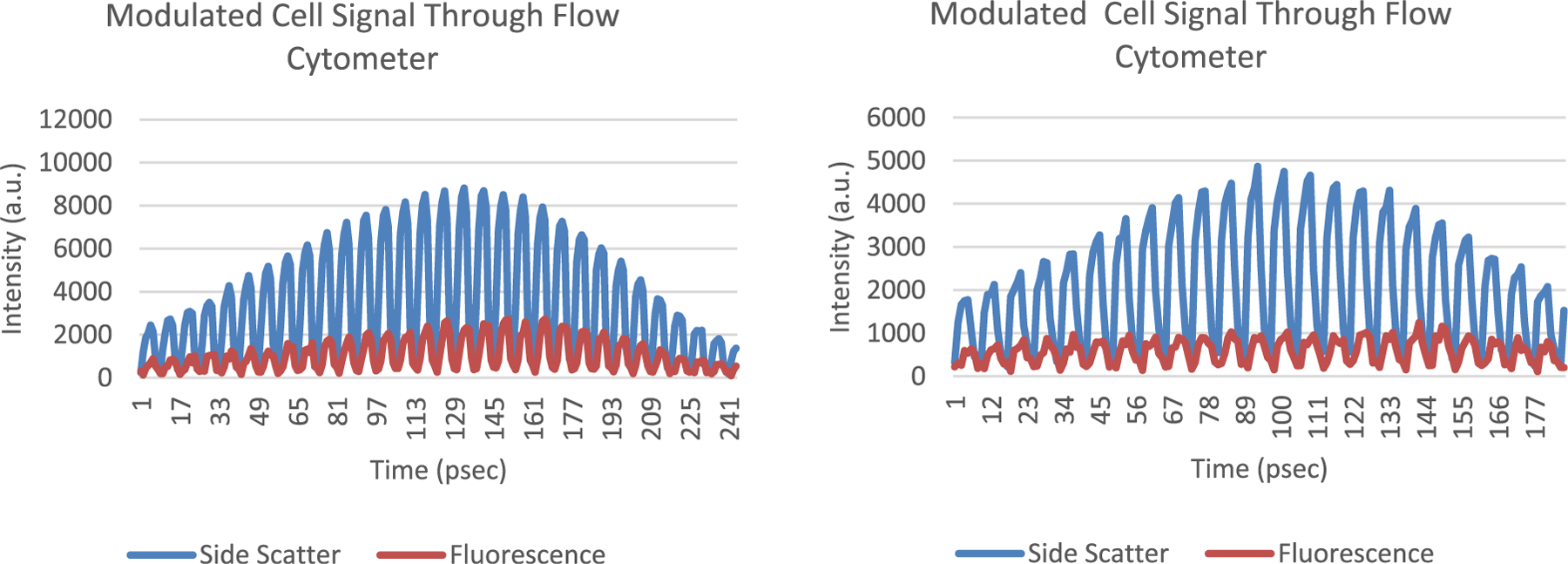
Two examples of cells progressing through the laser to present examples of the diverse populations of NAD(P)H within a single cell population.

For TRFC, data (i.e., cytometric waveforms) were analyzed for 200 cells (meeting the aforementioned criteria) over four independent experiments for both MCF-7 and MCF-7 TamR cells. TRFC data are also included in Fig. B.1. from TRFC measurements to present the variance of NAD(P)H within a single cell population. Figure 5 is representative data comparing the fluorescence lifetime for each cell type. The average fluorescence lifetime of NAD(P)H measured from MCF-7 TamR cells decreased by 0.3 ns compared to MCF-7 (Table 1). The mean fluorescence lifetime for each independent cell population for MCF-7 cells was 5.2 +/− 0.53 ns (standard error of the mean) while the mean fluorescence lifetime of the MCF-7 TamR cells was 4.9 +/− 0.46 ns. As discussed previously, the two-component fluorescence lifetime of NAD(P)H when weighted trends more toward the shorter lifetime is an indicator of a shift toward glycolysis from mitochondrial respiration (i.e., free NAD(P)H). The one-way ANOVA results showed a *p*-value < 0.05 between the fluorescence lifetime of MCF-7 and MCF-7 TamR samples and a post-hoc Tukey test confirmed a significant difference between the two groups for each experiment.

## 4. Discussion

Comprehensive studies of autofluorescence imaging for metabolic profiling describe and establish the relationship between the brightness of NAD(P)H and a cell’s corresponding metabolic profile.^59^ Yet it is widely reported that confounding factors influence the amount of autofluorescence detectable. NAD(P)H autofluorescence is affected by local pH, viscosity, temperature, free versus bound states, as well as cell density.^28^ Representative images are presented in Fig. 3; when processed for contrast appear similar and are included in this study to emphasize this point. That is, brightness is generally nonquantitative, and interestingly much of the literature has shown, over-simplistically, that when the autofluorescence intensity increases, the cell shifts its metabolism toward glycolysis (i.e., lower mitochondrial respiration).^59^ When the counts per-pixel were summed under the conditions of this study and by confocal imaging, a greater average raw integrated intensity from MCF-7 TamR cells in comparison to nonresistant MCF-7 cells resulted. The higher overall average intensity for the resistant cells compared to the sensitive cells agrees with expected trends in unbound NAD(P)H states (i.e., shift towards more active glycolysis). This also supports the results from the other two independent metabolic measurement methods (i.e., Seahorse and TRFC). Nonetheless, autofluorescence microscopy data are not an optimal way to determine the metabolic profile of the cells.

Seahorse analyses and metabolomics are examples of methods taken to identify metabolic features of tamoxifen treated and resistant cells. With our Seahorse analysis, we measured a shift in the metabolism of MCF-7 TamR cells toward glycolysis in production of ATP. Whereas, MCF-7 sensitive cells were found to rely more heavily on OXPHOS. This is supported by other work in which the inhibition of mitochondrial complex I has been attributed to decreased oxygen consumption and altered AMPK expression in breast cancer cells and isolated mitochondria.^17–19^ Reactive oxygen species, lactate, and ATP expression have also been shown to be comparatively different in resistant populations.^16–19^ In recent metabolomics work, it was found that pantothenic acid and beta-alanine were elevated in MCF-7 TamR cells,^21^ which are associated with glycolytic activity and cancer aggressiveness.^60^ Also found were endpoints of the TCA cycle (e.g., citric acid, succinic acid, and oxoglutaric acid) that were reduced in MCF-7 TamR cells, which was attributed to the Warburg effect.^21^ The data presented here build on this understanding using an ATP rate assay to correlate ATP generation preferences to metabolic cycles.

With TRFC we can uncover trends in measured shifts in the fluorescence lifetime of tamoxifen resistant populations (i.e., unbound state of NAD(P)H) and use the correlative Seahorse measurements to verify the glycolytic or OXPHOS profiles. We observed shifts in the autofluorescence lifetime and evaluated the signal-to-noise ratio to optimize the sensitivity of our TRFC system. We also calculated the average photon counts from a statistical photon calculation^57^ (Table D.1) and the excitation fluence (Table E.1). A high standard deviation of the fluorescence lifetimes with TRFC is attributed to variation in the number of cycles used for the phase calculation for any given waveform, the selected modulation frequency, the number of events averaged, as well as signal-to-noise. These parameters are also factors in the resulting absolute fluorescence lifetimes. Our resulting lifetimes, although within reported ranges, are longer than the decay times most commonly reported in FLIM.^31^ Other factors that affect this include different micro- and macro-environmental conditions of the cells. FLIM analyses are typically performed on viable, adherent monolayer cells compared to cell suspensions. Interestingly, it is well documented that the fluorescence lifetime is influenced by several environmental conditions such as pH, confluency, extracellular conditions, and other factors.^28^ Therefore, our goal is to optimize the TRFC and continue to validate with other analytical techniques.

Finally, it is important to note that NAD(P)H fluorescence lifetime analysis requires a deep UV interrogation which comes with other potential drawbacks if considering cellular damage and alteration to metabolism. Nonetheless, deep UV analysis is becoming more prevalent in flow cytometry with the demonstration of 266 nm laser excitation by Telford^61^ as well as commercial implementation in various systems (e.g., Cytek Aurora, Sony ID7000, and BD FACS Symphony A5). Reaching into the deep UV serves many purposes in cytometry both for autofluorescence spectral characterization as well as expansion of the wavelengths possible for multiplexing.

In future work, we intend to study both the ER-dependent mechanisms of resistance as well as improvements in the techniques used to evaluate resistance. These include establishing cell sorting based on the fluorescence lifetime, phasor analyses, and the design of a metabolic optical redox flow cytometer. As with many demonstrations of new cytometry systems, the sensitivity, resolution, and speed are critical and are important to continuously optimize.

## 5. Conclusion

A series of metabolic profiling experiments were performed to determine if transitions between OXPHOS and glycolysis are measurable for breast cancer cells that are resistant to the chemotherapeutic tamoxifen. This included demonstrating TRFC as a means to search for metabolic shifts cell-by-cell for moderately sized populations, which was the main goal of this study.

This work has an impact on the broader field of therapeutics and resistance because chemotherapies often act more effectively on dividing cells or are used as respiratory chain inhibitors. Therapies have particularly focused on this relationship because many cancers are characterized metabolically by the Warburg effect in which aerobic glycolysis occurs as well as an increased dependence on ATP to support rapid cell growth and division. Moreover, oncogenes that promote cell proliferation can alter the metabolic characteristics (e.g., promote glycolysis) of cells as a requirement for transformation. This is also complicated because cells that become resistant to targeted therapies are driven by these pathways. Therefore drug-resistant populations, for example, will depend on elevated glycolysis. Similarly, therapies that accordingly target glycolytic metabolism could be beneficial during the identification of resistance based upon our findings. While there is not a full understanding of the exact mechanisms in the metabolic pathways that are affected by resistance, our work supports other studies that correlate glycolysis with resistance. We add to this knowledge by demonstrating NAD(P)H decay times are related to the presented metabolism during prolonged cell culture in the presence of tamoxifen.

## Figures and Tables

**Table 1.** Individual 200 cell fluorescence lifetime assessment MATLAB data.

Experiment	1	2	3	4

	Average fluorescence lifetime (ns)			
MCF-7	5.5 ± 1.6	5.7 ± 1.6	4.5 ± 1.1	5.1 ± 1.7
MCF-7 TamR	5.4 ± 1.7	5.0 ± 1.5	4.3 ± 1.3	4.9 ± 2.0

*Notes*: Average fluorescence lifetimes of 200 events per experimental repeat per cell type as well as the standard deviation across the events is presented.

## Appendix D. Statistical Photon Count

The statistical photon count, $N_{P}$, was calculated based on the work of Giesecke *et al.* which utilized the coefficient of variance to evaluate the photon count. The coefficient of variance was calculated as the standard deviation/mean*100. The statistical photon count was calculated using the following equation^57^:

$$N_{P}={\left(\frac{100}{\text{CV\%}}\right)}^{2}$$

The CV was calculated from the maximum intensity (calculation process explained in the methods section) of the fluorescence channel measured for the SNR for both the MCF-7 and MCF-7 TamR cells for each independent experiment of 200 cells. The CV was then averaged for the four experiments

**Table D.1.** Statistical photon count.

Statistical photon count	
MCF-7	MCF-7 TamR
9	2

*Notes*: The statistical photon count evaluated for MCF-7 and MCF-7 TamR cells is presented in the table. The photon count is based upon the average coefficient of variance of the maximum intensity (height) for the cells within the experimental repeats in the fluorescence channel.

to calculate the CV for each cell subtype. The number of photons was then calculated using Eq. (D.1). The resulting statistical photon count is presented in Table D.1.

While this photon count is low, this is not unexpected. Autofluorescence is an inherently dim signal, but based upon the SNR and the photon count presented here, we are confident in our systems ability to resolve measurements of NAD(P)H.

## Appendix E. Laser Light Dose

Light Dose=Irradiance∗Transit time

$$=\frac{\text{Power}}{\text{Area}}*\text{Transit time}$$

Assuming the laser power is consistent across the beam:

$$\frac{60\text{ mW}*\frac{1\text{ W}}{1000\text{ mW}}}{1.984\;{\text{mm}}^{2}*{\left(\frac{1\text{ cm}}{10\text{ mm}}\right)}^{2}}*200\text{ps}*\frac{1\text{ sec}}{1x10^{12}\text{ps}}$$

$$=6.04x10^{-10}\frac{\text{J}}{{\text{cm}}^{2}}$$

The light dose limit at 375 nm is 25 J/cm^2^ and our systems light does of 3.04 × 10^−10^ J/cm^2^ is well below this limit.^58^

Also noteworthy is that this approximation does not consider the mode of the laser (i.e., Gaussian before any changes in collimation and lens shaping).

**Table:** Table E.1. Laser light dose.

Laser light dose at 60 mW
6.04 × 10^−10^ J/cm^2^
